# Optimization of an Optical Test Bench for Tire Properties Measurement and Tread Defects Characterization

**DOI:** 10.3390/s17040707

**Published:** 2017-03-29

**Authors:** Juan Jesús Castillo Aguilar, Juan Antonio Cabrera Carrillo, Antonio Jesús Guerra Fernández, Sergio Postigo Pozo

**Affiliations:** Department of Mechanical Engineering, Doctor Ortiz Ramos s/n, 29071 Malaga, Spain; jcabrera@uma.es (J.A.C.C.); ajguerra@uma.es (A.J.G.F.); spostigo@uma.es (S.P.P.)

**Keywords:** frustrated total internal reflection, tire, contact pressure, sensors

## Abstract

Tire characteristics and behavior are of great importance in vehicle dynamics since the forces transmitted in the tire-road contact are the main contributors to global vehicle performance. Several research groups have focused on the study and modeling of tires. Some of the most important factors that need to be known are tread characteristics and pressure distribution in the tire-ground contact patch. In this work, a test bench has been used to adequately determine the aforementioned factors. The measurement principle of the test bench is the frustration of total internal reflection (FTIR) of light. It makes use of a laterally illuminated glass on which the tire leans. An interposed plastic interface between them causes the reflection of light. Finally, a video camera captures the bright image formed through the glass. The brightness level in each pixel of the image is related to existing normal pressure. A study of the parameters that affect the test bench calibration such as type of interface material used, diffuse light, hysteresis, creep and transverse light absorption is performed. Experimental tests are conducted to relate tire inflation pressure and camber angle to the pressure distribution. Furthermore, the test bench is used to detect and evaluate the influence of defects in the tire on the contact pressures.

## 1. Introduction

The study of tire behavior is of great interest in the field of vehicle dynamics. The tire is the only contact zone of the vehicle with the road. The forces that are transmitted to the vehicle and cause its desired or undesired behavior are mainly those generated in the above mentioned contact zone. For this reason, a large number of tire models [1,2,3] have been developed to predict tire behavior. Most of these models require a series of parameters and tire characteristics to reproduce the forces that occur in them due to external solicitations. Some of the main factors that need to be known are the characteristics of the tire tread and its normal contact pressure distribution. These models are fitted using data obtained from test benches that simulate the contact between the road and the tire. There also exist models that focus on the road-tire interaction using data provided by the sensors installed on the vehicle testing the tires under real working conditions [4]. This way, the stiffness, roughness and thermal phenomena are taken into account in the forces estimation.

The interest in obtaining the tire tread and the pressure distribution in the contact zone is mainly due to two reasons. The first one is that these data are used to fit the pressure distribution to mathematical functions that can be used as input in simulation models. On the other hand, the determination of all parameters of the tire patch, such as contact area, size, pressure map, behavior of evacuation channels, etc. is of great help in optimizing the design of a tire.

Commonly used techniques to measure contact pressures are based on two different technologies:

- Systems based on piezoresistives or piezoelectric sensors [5,6,7,8,9,10]; and
- Sensors based on optical phenomena [11,12,13,14,15].

In the first technique, the variation of resistance with pressure (piezoresistive sensors) or the generation of electric current with the application of a force (piezoelectric sensors) of the measurement points located in the contact zone allow obtaining the pressure value at discrete points in the tire patch. It is a technique with a high cost and a low number of measurement points which produces low resolution pressure maps. In some cases, these data can be extrapolated to obtain an approximate map of pressures in the whole contact area. Therefore, this technique is limited to the study of very simple treads with a low number of measurement points in the patch.

The second technique is based on the physical phenomenon of Frustration of Total Internal Reflection (FTIR). Chodera [10] was the first author who proposed a sensor based on this phenomenon. According to this author, by placing a thin plastic interface on a transparent surface previously illuminated with light inside, bright spots under the glass when applying pressure to the plastic could be obtained. The brightness of these spots is related to the applied pressure.

Later, Betts and Duckworth [12,16] applied this technique to biomechanics tests. FTIR was used in such a way that they could obtain the pressure distribution in the foot-ground contact. The image was collected by the camera through a mirror. C.R. Gentle [13] carried out a study about the application of this optical technique to the measurement of pressure distribution in the contact between tire and road. In this work a test bench was developed in which the pressure distribution was measured with the tire under uncontrolled vertical load and without the capability of changing neither the camber nor the slip angle.

Simultaneously to the development of optical techniques, the techniques based on piezoresistive and piezoelectric pressure sensors were improved, increasing the resolution of the grid where they were inserted. Sakai [5] obtained values of the normal and tangential pressures in the tire–road contact zone in static conditions using piezoresistive pressure sensors.

Optical techniques provide the best spatial resolution in the mapping of pressures even with the increase in the number of piezoresistive or piezoelectric sensors used in the grid.

Research carried out by C.R. Gentle [17,18] as well as Chacko [19] focused on the influence of the plastic interface due to its importance on the direct measurement of the pressure in the contact zone, as well as creep and hysteresis phenomena. They also evaluated the influence of the composition of the plastic on its mechanical properties and its relationship to its optical behavior.

In all cases, the contact patch is affected by the installation of the piezoelectric sensors or by the presence of the plastic lamina in the optical measurement systems. Therefore, the main drawback of this kind of systems is that they do not test the tires in real world conditions. On the other hand, the main advantages are that they allow more controlled conditions, enable the study of the influence of one or several variables independently and increase the speed in obtaining results, making it a powerful tool to understand tire behavior.

More recently, the Mechanical Engineering research group of the University of Malaga developed a test bench to obtain and study the pressure distribution in the contact patch of the tire [20]. The pressure map is obtained by making use of the FTIR phenomenon. The calibration process of the interface plastic layer is automated allowing optimizing the grey levels of the obtained image and thus improving the reliability of the obtained pressure map. The developed bench also allows studying the influence of the camber angle variation and the tire load in a controlled manner. This way, in-depth studies can be carried out on how the aforementioned parameters affect the pressure distribution in the tire-road contact.

The objective of this work is to develop a methodology to improve the results obtained in previous studies to assess the influence of some factors in the process of obtaining an image representative of the pressure distribution. In order to do so, we have deepened the study of factors related to the use of the test bench, such as: camera settings, system calibration, study of the materials to be placed in the interface, creep and hysteresis influence. This research allows us to design a test methodology that improves the image quality and the reliability of this technique. The image processing allows obtaining mathematical models to relate factors of influence in the use of tires to the pressure distribution and the characterization of tire defects.

This paper first describes a test bench to measure tire contact patch pressures based on the FTIR phenomenon, which is introduced in Section 2. Factors affecting image capture and the system calibration process are described in Section 3. Section 4 includes some tests conducted to evaluate the influence of tire inflation pressure and camber angle on the pressure distribution. An interesting field where the test bench has been proved to be a very useful tool is the detection and characterization of failures in the tire belt. Some case study examples are included in Section 5 and Section 6. Section 7 concludes this paper.

## 2. Test Bench

The test machine is based on the optical phenomenon of Frustration of Total Internal Reflection (FTIR). Total Internal Reflection (TIR) of light can only occur when light travels through a medium and strikes the interface with another medium with a lower refractive index. For example, it will occur with light reaching air from glass, but not when reaching glass from air. According to Snell’s law, if the incident angle is greater than the critical angle (*θ*_c_) the light beam do not pass through and is totally reflected. The critical angle is derived from Snell’s law (1):

(1) $$\gamma_{1}\cdot sin(\theta_{1})=\gamma_{2}\cdot sin(\theta_{2})$$

where *γ*_1_ and *γ*_2_ are the refractive indexes of medium the light is propagating and the medium the light at the boundary, respectively. *θ*_1_ is the incident angle and *θ*_2_ is the angle of the refracted light beam. The critical angle is derived by setting *θ*_2_ = π/2, so that the refracted light beam would travel along the surface of the boundary.

**Figure 1:**
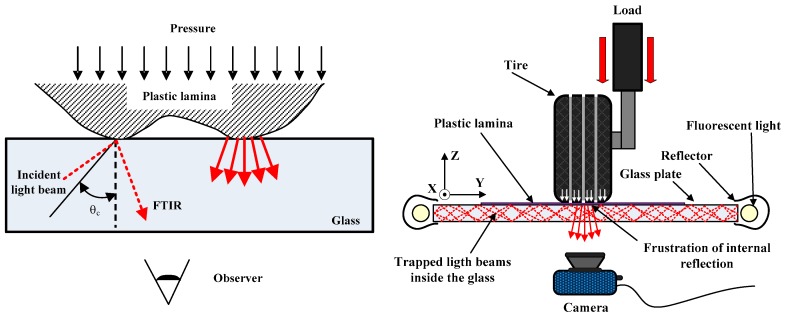
FTIR phenomenon (**left**); and test bench scheme (**right**).

However, if a plastic lamina is in contact with the glass, TIR can no longer occur since plastic has a higher refractive index than glass. When the plastic lamina comes in contact with the glass, the internal reflection of the light gets frustrated at that point and bright luminescent spots are created. This frustrated light then escapes the medium and is scattered downwards. This scattered light can be observed and captured with a camera Figure 1 (left).

The measuring system consists of a 12 mm thick glass that is illuminated from its sides by fluorescent tubes. A plastic lamina is placed between the tire and the glass. A more intimate contact with the glass is favored when the lamina is deformed by the tire. The image of the patch is collected by a camera to be further processed by a PC Figure 1 (right).

As mentioned before, the optical phenomenon the test machine is based on and which allows the tire contact pressures to be obtained is called frustration of total internal reflection. FTIR occurs when the internal reflection of the light that is trapped within the interior of the glass is interrupted. The light rays are deviated projecting towards the outside of the glass which causes the plastic to illuminate.

**Figure 2:**
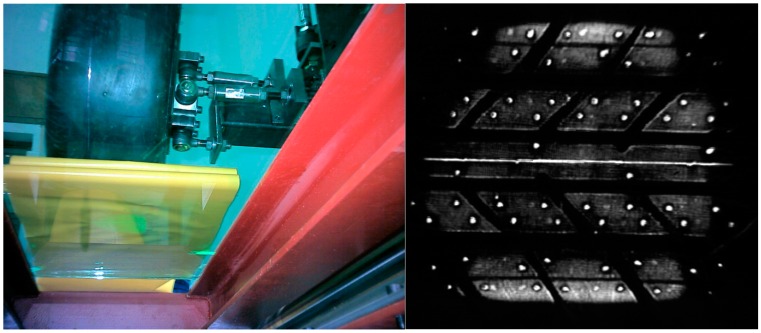
Test bench bottom view (**left**); and example of image obtained with the test bench (**right**).

The plastic has a rough appearance at a microscopic scale. This causes the plastic to gradually deform as the applied pressure increases causing a more intimate contact between plastic and glass and providing a greater number of contact points where the total internal reflection of the light is interrupted. Consequently, the number of bright spots grows in a given area as the pressure increases. Due to the increase of these bright spots, the overall brightness level of the area will increase in proportion to the pressure. This way, an image is obtained in which the points of highest pressure appear brighter (Figure 2).

The calibration of the camera is carried out by capturing an image of a grid of known dimensions. This image is later processed by the computer to correct the distortion introduced by the curvature of the lens and to obtain the pixel–millimeter relationship. The position of the camera, the zoom and any other camera related factor remain constant in the tests.

The camera provided files with RGB images. Images were captured as grayscale images, so the value of the three RGB components of each pixel should have been the same. Some noise was observed, which led the three components not to have exactly the same value. Consequently, the Grey Level (GL) of a pixel was defined as the mean value of the three RGB components of that pixel to reduce disturbances.

**Figure 3:**
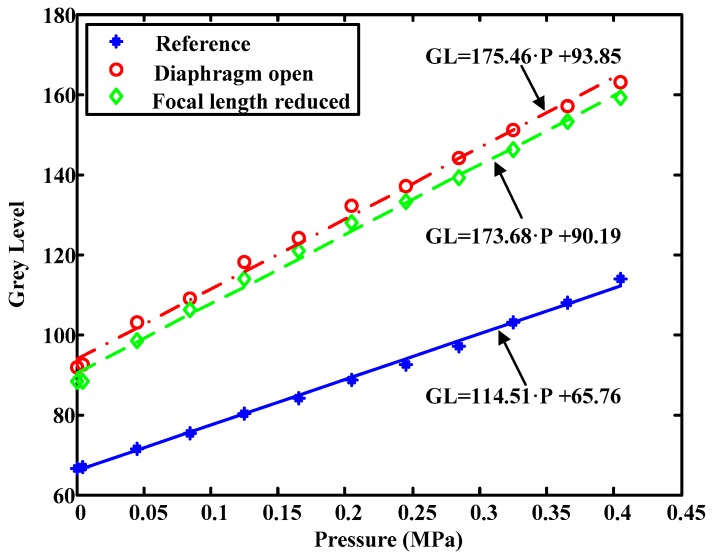
Influence of the diaphragm aperture.

Graphics file formats store RGB images as 24-bit images, where the red, green, and blue components are 8 bits each. The minimum and maximum value for each component is 0 and 256. Therefore, in this application, the theoretical range of the GL is (0–256), meaning 0 is a completely black pixel and 256 is a white pixel. However, the real GL range was obtained during the calibration process. As can be seen in Figure 3 and Figure 5, the real GL range was influenced by the existence of the interface lamina, the illumination conditions and camera related factors.

The use of specially designed parts is required for the interface material calibration. The calibration parts present a cavity in their interior, which allows the entrance of pressurized air that will be in contact with the interface material. This air exerts equal pressure on all its walls. The part is placed over the material to be calibrated, which becomes the last one of the walls of the cavity. This way, the existence of uniform pressure on the calibration zone is ensured. Then, air pressure is increased from minimum to maximum calibration pressures. Images are captured at regular pressure intervals. Thus, a series of characteristic images of each one of the applied pressure values are obtained. For each image, a Gaussian distribution with all the present grey levels within the region of interest is calculated for each image. The average grey level of this distribution is the value associated to the applied pressure. This way the interface material calibration is reached, fitting the obtained values to a polynomial of a suitable degree. A more extensive description of the test bench and camera and interface material calibration can be found in [20].

## 3. Study of Factors with Influence on the Image Obtaining

There are controllable and non-controllable parameters in the process of image capture. The following are uncontrollable factors: the absorption of the crystal in the transverse direction and the influence of bright points on the grey measure of adjacent points (which influences the contrast). These parameters are studied to evaluate their influence on the obtained image. Tests show that they do not significantly alter the obtained results, provided that the test machine has been designed in a suitable way.

Controllable parameters are mainly due to environmental factors external to the test bench, such as temperature and ambient light, whose influence was studied in [20]. These will be held constant to minimize their influence on the capture of images. Other controllable parameters are those due to the camera used, such as focal length and diaphragm aperture.

### 3.1. Camera Related Factors

This section is devoted to evaluating the influence of the degree of diaphragm aperture and focal length on the images obtained in the test bench. The tests are performed keeping the ambient lighting of the room constant.

The test is carried out in three steps. First, a base image is obtained in which the camera is adjusted to its default setting. Then the diaphragm aperture is increased, which causes the amount of incident light to be greater, and a new image is captured. Finally, with that new diaphragm aperture, the focal length is modified and, consequently, the focus to have a clearer image again.

It can be observed in Figure 3 that the variation of diaphragm aperture affects the amount of incoming light in the camera but also its sensitivity, causing either an increase or decrease in the ordinate at the origin of the calibration curve as well as a variation in its slope. In all figures in this paper, the units of each coefficient of the fitted polynomials, when included in the figure, are GL/P^n^, where n stands for the nth coefficient of the polynomial. Likewise, R is the correlation coefficient.

In addition, it can be seen that there is hardly any variation between the calibration curves obtained varying only the focal length. The deviation in the slopes is about 1%, so they can be considered parallel. Likewise, a slight vertical displacement of the curve can be observed by varying the focal length.

Specifically, the focal length has been decreased in the last sample, i.e., the image size has been decreased. If this is so, the amount of light that the camera receives is greater causing the calibration curve to move upwards, which actually occurs. The ordinate difference in the origin is of the order of 4 grey levels approximately, that is, the effect is less than if we illuminate the room.

Therefore, the tests are carried out by setting the parameters of the camera so that an image is obtained in optimal conditions for its analysis. These parameters are kept constant throughout all the tests.

### 3.2. Plastic Lamina Selection

The importance of selecting the proper plastic interface is of high relevance for the study of image. The main goal when choosing the plastic lamina is that the resulting image is composed only of levels of grey suitable to the pressure levels in the contact patch and that it provides a clear image. This way, conclusions can be drawn qualitatively, with the simple inspection of the pressure distribution. However, if a pressure quantitative analysis is to be made, a known pressure–deformation relationship must exist.

The main plastic lamina characteristics that should be to be known before any test is conducted and that determine their selection are:

- Color
- Texture
- Thickness
- Sensitivity
- Linearity

Color and texture influence the quality of the obtained images. The thickness mainly affects the hysteresis of the material. Linearity and sensitivity depend, as it is shown below, fundamentally on the material of the lamina used. Two different plastic laminas have been tested from low to high pressures to evaluate the range in which the materials have a linear behavior.

### 3.3. Plastic Lamina Mechanical Saturation

The mechanical saturation of the plastic is reached once the contact between plastic and glass is the maximum possible. At this time, however much the pressure increases, the grey level will not increase.

The importance of studying the mechanical saturation of plastic resides in determining the area in which the plastic stops behaving linearly and its sensitivity decreases. Two experiments were carried out to explore the linearity and sensitivity of two plastic laminas. Sample 1 was a PVC white lamina while sample 2 was a thin green translucent plastic lamina.

**Figure 4:**
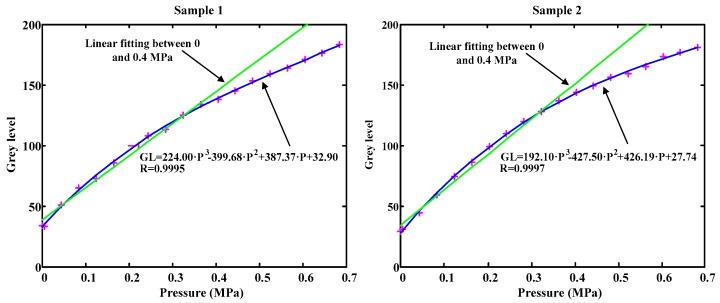
Plastic laminas mechanical saturation.

Figure 4 shows how, as the pressure increases, the tendency of the plastic moves away from its linear behavior. It is also observed how this phenomenon begins to appear from 0.4 MPa. This pressure will be established as a limit in all the calibrations. However, the whole data in each test can be appropriately adjusted by a cubic function. This way, the whole range of pressures could be used. By limiting the calibration to 0.4 MPa the linear approximation can be used as long as the measured pressure range is below that value.

### 3.4. Samples Comparison

**Figure 5:**
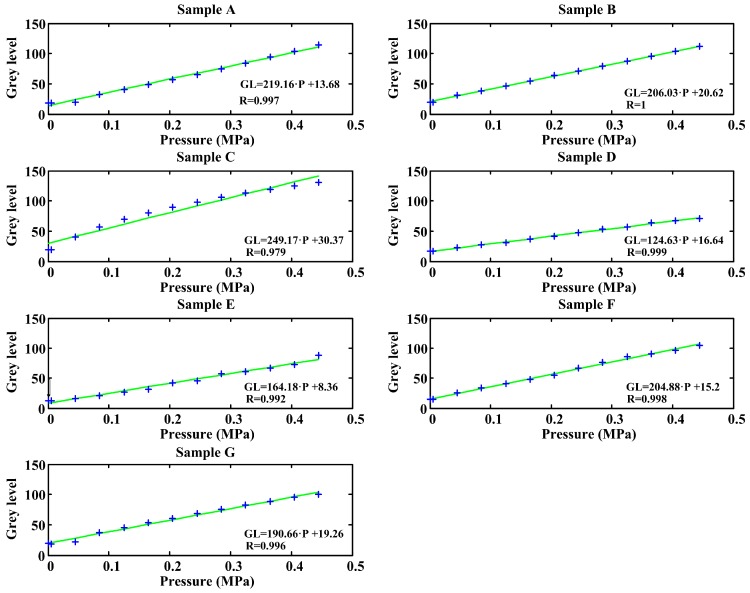
Samples calibration.

The plastic interface is the component with the highest influence in the quality of the images. Therefore, a proper plastic interface with an optimum performance depending on the tests to be performed has to be chosen to carry out the experiments. For this reason, different samples have been calibrated in order to select a plastic with linear behavior in a sufficiently wide range, with an adequate sensitivity and a fine enough texture so that the captured image is representative. Figure 5 includes the results of the seven samples tested. The most relevant comments and conclusions about each sample are included next.

- Sample A: White opaque plastic. The texture is very fine not appreciating any protrusion at any point and being homogeneous throughout the surface. A good correlation is obtained although there are slight fluctuations at low pressure levels that make it have poor linearity in that range. Among all the samples, it has the finest texture, so it will be the most interesting sample to obtain high quality images from although it can introduce more error than other samples for quantitative analysis.
- Sample B: White opaque plastic. The texture is of greater thickness, although not excessive and has a superior brightness to the previous sample. The sample thickness is also slightly bigger. The obtained data are perfectly adjusted to a straight line for all pressure values. This sample is interesting because it has a very high sensitivity and a very good correlation coefficient.
- Sample C: Yellow plastic. The texture is very fine on the plastic face and rough on the textile side. It is clear how the behavior of this plastic is not linear but is most approximate to a second order curve. For this reason this sample will be discarded for use.
- Sample D: Silicone opaque, thicker than sample A. This sample is slightly stiffer than the one above. It presents a correct linear behavior. It is therefore also suitable to be used in tests.
- Sample E: Light blue plastic on one side and dark blue on the other. It is the sample of greatest thickness, approximately 1 mm, and it has a very fine texture. The image obtained with this sample does not provide good definition because of its great thickness. However, it produces images with great homogeneity.
- Sample F: Yellow PVC sheet, opaque and with a slight thickness. Both sides have been tested. The smoother face did not show good behavior so only the results for the matte face are shown. Although it has very high sensitivity, it has a greater dispersion than other samples.
- Sample G: White plastic with a cotton component on one side and completely smooth on the other one. The thickness is higher than in the previous case but not as large as in sample E. The smooth face has been tested. The results have not been satisfactory because of the textile component. Despite being on the opposite side, it influences the homogeneity of the image.

**Figure 6:**
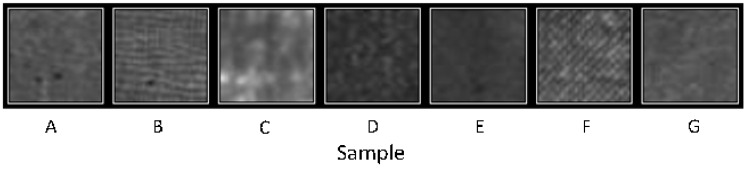
Samples textures.

Some samples have been discarded because they did not fit the linear behavior. However, we must also consider its texture and sensitivity when choosing the right material. Although sensitivity can vary by modifying the parameters of the camera, the texture is an inherent property of each plastic. Figure 6 shows a 5 × 5 mm constant pressure region of images taken in the calibration process of each of the samples.

The image of sample C is somewhat non-homogeneous due to its textile components. Sample F, which in previous studies was the one that presented better results, now shows the roughness of its surface in an evident way. Sample D has very low sensitivity, the lowest of all samples. Sample E is not suitable due to its thickness. Samples A, B and C are suitable for the tests. Although some roughness can be observed in the image obtained with sample B, this sample had the best performance in the tests. In addition it is the material that exhibits the most linear behavior, fitting perfectly to a straight line and also having a fairly high sensitivity. For these reasons sample B was selected to be used in the tests.

### 3.5. Diffuse Light

The phenomenon of total frustration of internal reflection of light shows that the light beam leaves the glass in which it was confined to illuminate the plastic interface at the points of contact. Once the beam of light comes out of the glass, the plastic interface absorbs one fraction, lets another pass through, and if it is clear, it reflects a large amount of it. What happens with the reflected rays is studied below.

To answer this question, it would be necessary to know the angle of incidence of the ray in the crystal at the point of contact. The optical properties of the interface (crystal-plastic) have to be known to determine the deviation of the angle that occurs when the reflection is annulled (refraction). These data are used to determine the angle of incidence of the light ray in the plastic.

The glass-air interface has a critical angle for the total internal reflection of approximately 42° with a refractive index of the glass (*γ_g_*) of around 1.5. Above this angle, the light is confined to the glass. The light is refracted at an angle close to 38° when the plastic lamina (with a refractive index *γ_p_* ≈ 1.6) is placed above the glass following the laws of refraction:

(2) $$\gamma_{g}\cdot sin(\theta_{g})=\gamma_{p}\cdot sin(\theta_{p})$$

The beam of reflected light may well come out at a greater angle than the critical one and re-travel on the glass, in which case its reflection may be frustrated at another point. However, it will also come out at an angle smaller than the critical one, in which case it will cross the glass and will be captured by the camera. In both cases, the contribution of that fraction of light that does not travel directly to the camera is global and will affect similarly to all the contact points. Therefore, its influence is not considered relevant.

**Figure 7:**
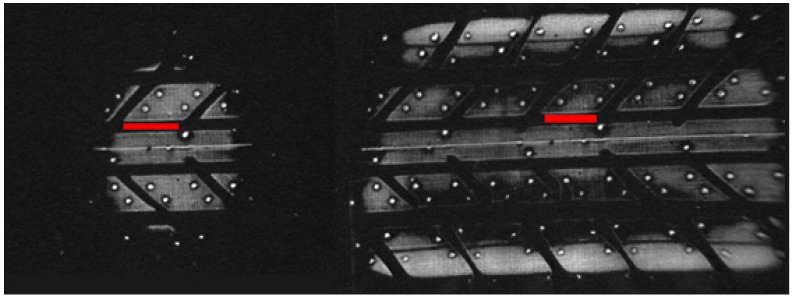
Diffuse light: unloaded tire (**right**); and loaded tire (**left**).

Two images of a tire have been taken to check the effect of diffuse light (Figure 7). The first image is obtained without load (only own wheel weight). The vertical load on the tire is 2000 N in the second image. A dark area that appears in both cases (central zone—red line) is selected for its study.

After performing several measurements of the grey level in the red region, it is observed that the mean grey level for the unloaded tire patch was 31.79 while for the loaded tire patch was 31.25. Thus, the grey level of an unloaded zone is not influenced by the proximity of a loaded zone.

### 3.6. Hysteresis

The ideal linear behavior of the plastic lamina that acts as an interface is altered when a load-unload test is carried out. It is observed that, although the load process follows a linear trend with a very high correlation factor, the hysteresis phenomenon appears during the unload phase. The developed software is capable of conducting tests to evaluate the hysteresis and to determine a hysteresis factor defined as:

(3) $$f_{h}=\frac{\begin{array}{cc} Load & area \end{array}}{\begin{array}{cc} Unload & area \end{array}}$$

**Figure 8:**
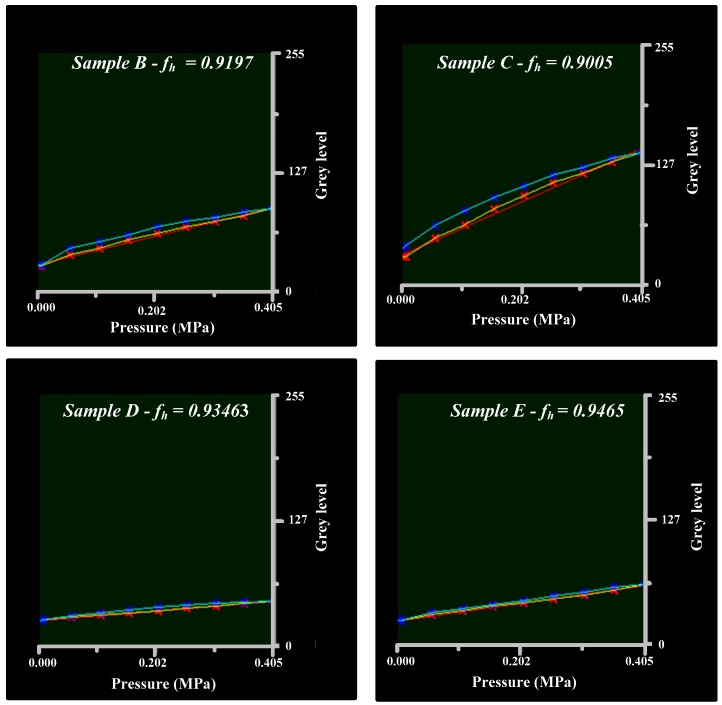
Hysteresis in samples B, C, D and E.

The factor will be equal to the unity in the ideal case. As it can be seen in Figure 8, sample C does not have a good hysteresis factor. The slope of the unload curve (cyan) is accentuated as lower values for the pressure are reached, coinciding with results obtained by Gentle [13].

It is observed that plastics with higher sensitivity have a higher hysteresis factor. This is because these samples are more elastic, with a greater amount of plasticizer causing them to be less effective in recovering the original state after being deformed. Stiffer plastics are less sensitive but their hysteresis factors are lower. The hysteresis factor will be of great importance in load–unload processes such as dynamic tests.

### 3.7. Creep Behaviour

Another dynamic phenomenon that affect the plastics used as interface is the influence of the time of exposure to pressure in the image obtained. Tests were conducted to evaluate how this creep behavior influence the measurements. To do so, the mean grey level at a constant pressure in the calibration piece was measured and plotted against time. Two tests at different pressures were carried out. The pressure in the chamber of the calibration part was kept constant at 0.20 MPa in the first test. Similarly, a second test was conducted at a pressure of 0.35 MPa. The results obtained can be seen in the figure below. As it can be observed, a phenomenon of material creep occurs as the time of exposure to the load increases. The figure shows that the evolutions of the grey levels in both tests are similar. As mentioned before, the experiment was carried out with two different loads in order to illustrate how this phenomenon varies when the load is modified. Another conclusion that can be drawn from the graph is that, as time passes, the creep speed of the plastic decreases.

**Figure 9:**
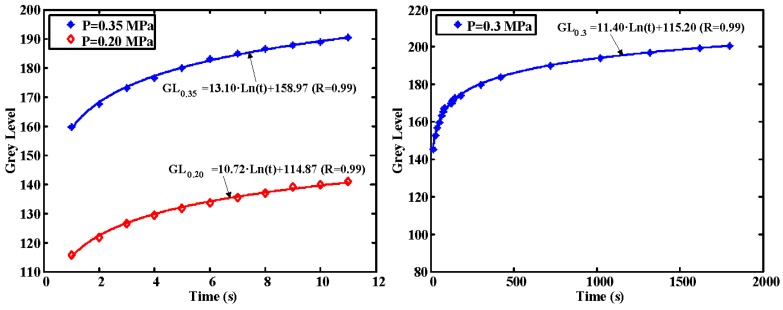
Grey level vs. time (creep). Short-time pressure exposition (**left**) and long-time pressure exposition (**right**).

A third test at a pressure of 0.3 MPa was carried out to explore the behavior of the material to long-time pressure exposition. In this test, the time interval between captures of images and the total time of the test was increased. The results are included in Figure 9. In all cases, the grey level vs. pressure relationship can be properly fitted with a logarithmic curve. In the following figures GL0.35, GL0.3 and GL0.2 stand for the grey level obtained at a pressure of 0.35, 0.3 and 0.20 MPa, respectively, and t is the time of exposure in seconds. Therefore, it seems that the grey level of the image tends to be stabilized after a long period of time.

**Figure 10:**
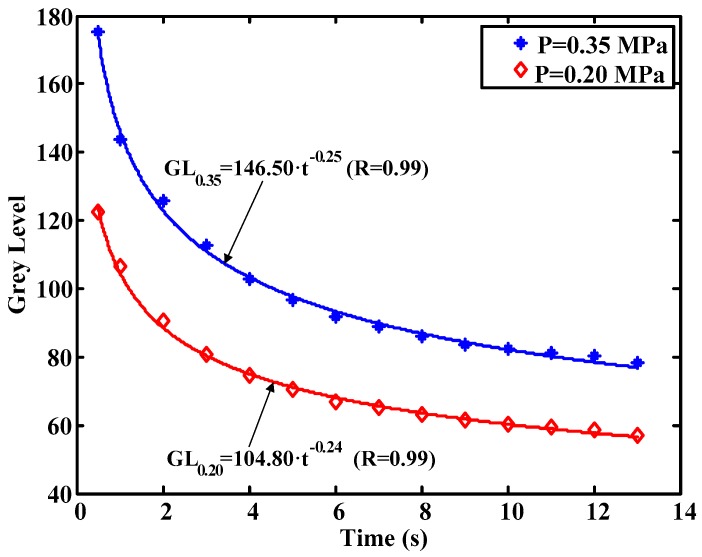
Grey levels during the unload phase.

A further test was conducted to check the behavior of the material in the unload process. In this case the test was carried out by unloading the material and measuring the time it takes for the pressure effects to disappear. As it can be seen in Figure 10, the grey level returned to its default values after 13 s approximately.

Previous tests provide fundamental information about the influence of the dynamics of the material in the process of obtaining the pressure-grey level relationship. As can be seen, the grey level depends on the time the picture is taken. Therefore, the pressure exposure time has to be taken into account for the proper calibration of the system. Not only is it necessary to record ambient calibration conditions, but also the pressure exposure time of the plastic to obtain the vertical load in the tire contact patch from the captured image. Therefore, the programmed algorithms to obtain the pressure take into account the exposure time using expressions similar to those included in Figure 9 and Figure 10.

### 3.8. Transverse Absorption of Light in the Glass

An important parameter to consider is the fact that the light intensity for a constant pressure may not be the same throughout the glass. Studies have been carried out on this phenomenon [12,13] finding that there is a relationship between the light intensity on the glass as the distance to the light source increases. It has been found that the ratio in the luminous intensity falls exponentially as the distance from the source increases if one single source of light at one end of the crystal is used. The decay in the intensity depends mainly on the transparence of the glass.

(4) $$I_{y}=I_{o}\cdot e^{-0.15\cdot Y}$$

(5) $$I_{y}=I_{o}\cdot e^{-0.0059\cdot Y}$$

where *I_y_* is the intensity at a distance *Y* (in mm) from the edge of the crystal and *I*_0_ is the intensity at the edge of the crystal. The unit of the damping constant in previous equations is 1/mm. The expression of the light absorbed on the glass is given by the superposition of the effect of each source if it is illuminated on both extremes, i.e., one light source on each side, that is:

(6) $$I_{y}=I_{o}\cdot e^{-0.15\cdot Y}+I_{o}\cdot e^{-0.15\cdot \left(a-Y\right)}\;(a=glass\;width\;in\;mm)$$

(7) $$I_{y}=I_{o}\cdot e^{-0.0059\cdot Y}+I_{o}\cdot e^{-0.0059\cdot \left(a-Y\right)}$$

**Figure 11:**
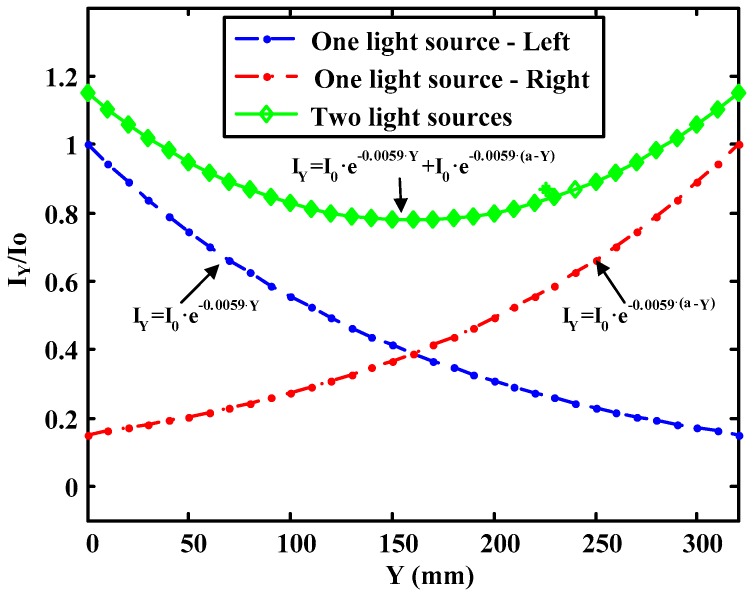
Transverse absorption of light vs. source/s location.

Equations (4) and (6) are obtained from reference [12] and Equations (5) and (7) from reference [13]. By observing both expressions, it can be verified that attenuation of the light is different in each case. This is due to the fact that the glass used or the incident light was different in both cases. As an example, Figure 11 shows the attenuation of the light in the glass when illuminated by only one source on each side of the glass and on both sides according to the second of the previous equations (*a* = 320 mm, *I*_0_ = 1).

**Figure 12:**
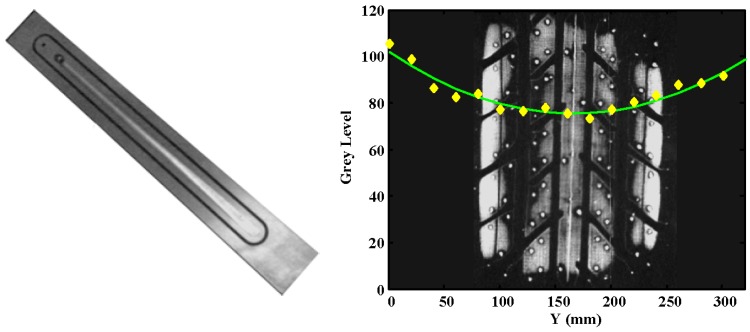
Calibration part (**left**); and transverse absorption of light vs. distance in the glass of the test bench (**right**).

A test was carried out to evaluate the influence of the transverse absorption of light in the glass in the calibration of the measurement system. Thus, the glass installed in the test bench was illuminated on its sides by means of two fluorescent bulbs. A specific part was designed to test the attenuation of the light in the glass of the test bench. This part is depicted in Figure 12. Grey values throughout the glass were measured every 20 mm when a constant pressure on the part was applied.

Behavior similar to the one shown in previous studies is observed. The measured grey levels (*GL*) vs. distance (*Y*, in mm) data are fitted to a similar mathematical function (Equation (8)):

(8) $$GL(Y)=83.43\cdot e^{-0.004955\cdot Y}+83.43\cdot e^{-0.004955\cdot \left(320-Y\right)}\;\begin{array}{c} \left(R=0.91\right) \end{array}$$

Figure 12 (right) shows the obtained experimental data (yellow diamonds) and the fitted curve (green line). There are around 25 grey levels of difference between the maximum (edges) and minimum values (center). However, the change in the grey level in the zone where the tire is placed (±10 cm around the center) is much smaller. The influence light absorption of the glass is less than 5%. Therefore, this value is small enough in the work area to not consider the effect of transverse light absorption in the glass.

## 4. Experimental Application

A series of tests have been carried out in which two independent parameters have been modified: camber angle and tire inflation pressure. The external parameters that could influence the measurement, such as luminosity and temperature, have been controlled. These tests were carried out with a Nankang EX601 165/65 R13 77H radial tire (Nankang Rubber Tire Corporation Ltd., Taipei, Taiwan) with a vertical load of 1000 N.

### 4.1. Contact Area vs. Inflation Pressure and Camber Angle

The maximum grey level of an unloaded zone was established as a criterion to assume contact. Images were filtered to remove noisy pixels. The contact area was obtained by adding the area of all pixels with a grey level above that value. It is worth mentioning that the area that corresponds to a pixel depends on the position of the pixel in the image. For this purpose, the area assigned to each pixel was previously obtained during the calibration of the image recording system (typically around 0.19 × 0.19 mm^2^).

**Figure 13:**
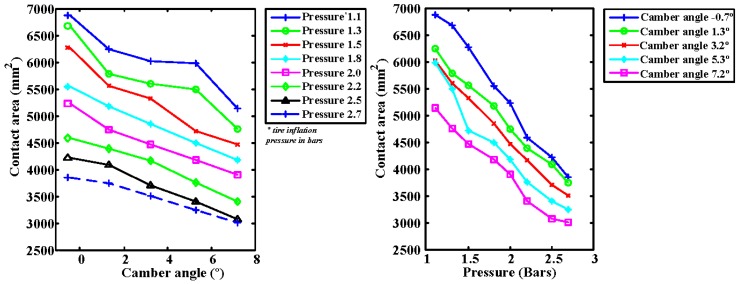
Contact area: vs. camber angle (**left**); and vs. tire inflation pressure (**right**).

The area of the contact patch has been measured as a function of tire inflation pressure and camber angle. Figure 13 (left) shows that, as the camber angle increases, the contact area decreases. The reason for this behavior is the change in the distribution of tire contact pressures. Figure 13 (right) shows that, as expected, when the inflation pressure decreases, the contact area increases. It can also be seen that the maximum area curve is produced for zero camber angles, i.e., when the wheel is perpendicular to the contact surface.

**Figure 14:**
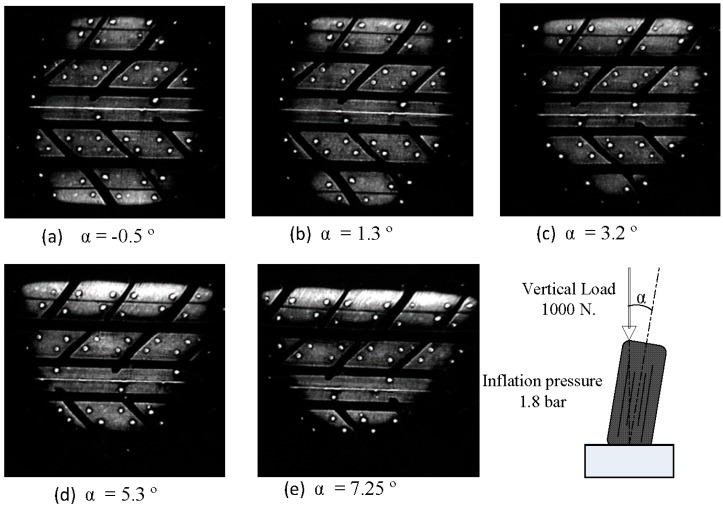
Contact patch at various camber angles.

As the angle increases, the tire lies more on the shoulder, as shown in Figure 14, where there is greater pressure due to the deformation suffered by the tread. This increase in pressure on the shoulders causes, if the load does not vary, a decrease of the area so that the equivalent vertical load is constant. The images obtained in the variation process of the tire camber angle are included in Figure 14 in order to verify this fact.

**Figure 15:**
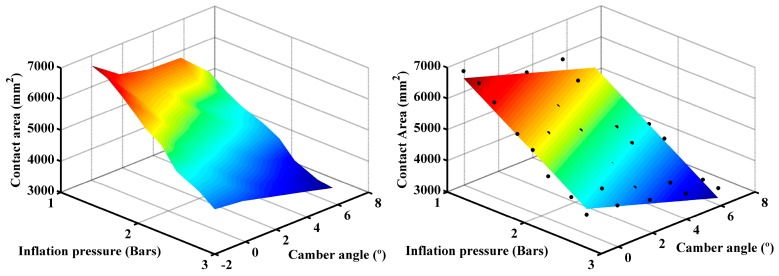
Contact area vs. camber angle and tire inflation pressure: measured data (**left**); and fitted data (**right**).

We can verify these conclusions by making a three-dimensional representation of these variables Figure 15 (left). It can be seen that the area gradient increase goes from points with large camber angles and high inflation pressures to points with zero camber angles and low inflation pressures.

It has been verified that the tire behavior is adequately adjusted to a linear model. This relationship has been obtained by least square fitting. Through the linear regression we obtain the following correlation:

(9) $$A\left({\mathrm{mm}}^{2}\right)=-174.5\cdot \alpha -1630\cdot p+8345$$

where *A* is the contact area in mm^2^, *α* is the camber angle in degrees and *p* is the tire inflation pressure in bars. As can be seen in Figure 15 (right), this expression fits adequately the experimental data (dots).

### 4.2. Center of Pressure vs. Inflation Pressure and Camber Angle

**Figure 16:**
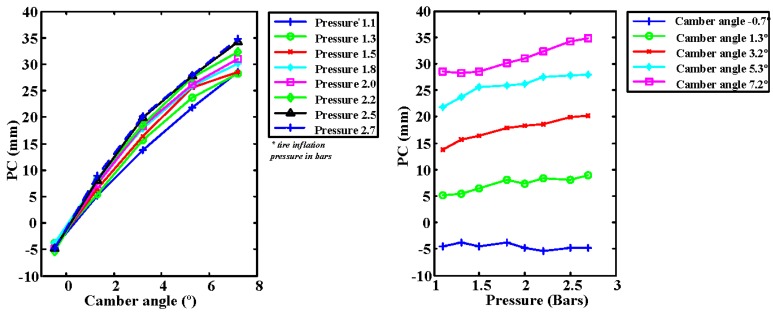
Pressure center (PC): vs. camber angle (**left**); and vs. tire inflation pressure (**right**).

The behavior of the pressure center of the tire as a function of the tire inflation pressure and the camber angle is evaluated next. The pressure center is the point where the force resulting from the surface-tire interaction can be assumed to be applied. This pressure center lies approximately on the symmetry axis of the tire patch for zero camber angle. However, there is a displacement of the pressure center towards the side on which the wheel is tilted for non-zero camber angles. The symmetry axis of the tire was taken as reference for the measurement of displacements (in mm) so that a zero displacement corresponds to a point located on that axis. The results are depicted in Figure 16.

It can be seen that the tire inflation pressure practically does not influence the position of the pressure center for null camber angles because the symmetry is maintained. However, as the angle increases, the deformation suffered by the tire depends on the inflation pressure, which causes it to influence in the displacement of the pressure center.

**Figure 17:**
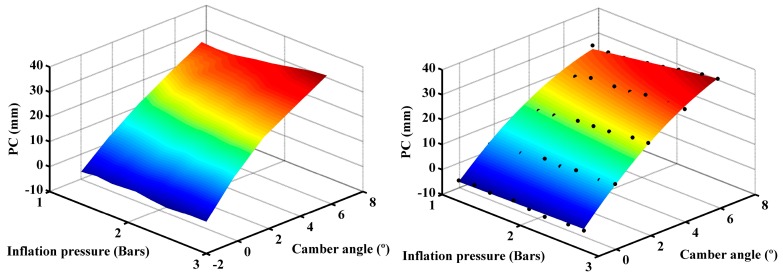
3-D representation of the pressure center: measured data (**left**) and; fitted data (**right**).

It can be observed from Figure 17 (left) that the increase in the gradient of the pressure center is related to the increase in the inflation pressure and, to a greater extent, to an increase of the camber angle. In this case, the relationship between the pressure center and the inflation pressure and camber angle is best fitted with a non-linear equation with the following form:

(10) $$COP=-2.582+0.801\cdot p+5.765\cdot \alpha -0.3351\cdot \alpha^{2}$$

where COP is the position of the center of pressures in mm, *p* is the pressure in bars and α is the camber angle in degrees. This last equation fits correctly the experimental data (dots). Therefore, it can be used to predict information that was not obtained experimentally.

## 5. Tire Defects Characterization

An interesting field where the test bench has been employed is to analyze how the existence of tread defects alters the contact area of the tire and the pressure distribution. To do so, tires were damaged with different types of defects.

One of the priority objectives of tire manufacturers is to achieve a tread as homogeneous as possible. This objective has been one of the reasons for the replacement of tires with a diagonal structure by tires with a radial structure in automobiles. The radial structure has, among other characteristics, a much more homogeneous contact patch since the tread is more rigid. For this reason, any defect in the tread that produces important alterations in the distribution of tire pressures is considered as detrimental to correct tire behavior. Likewise, defects that diminish the contact area are also worrisome.

A Hawk 700195/60 R14 radial tire model by Firestone was used in these tests. This tire had already been rolled and its tread presented a homogeneous wear. The tire was divided into 9 sectors, some of which were left undamaged to have reference patterns. Several images were taken before and after the damage for all the defects. In some cases, images were captured after having repaired the damage.

The images were taken with a camber angle of 0° and at a tire inflation pressure of 2.0 bar. The vertical load was 1000 N. The laboratory temperature was 24 °C and there was no ambient illumination.

### 5.1. No Defect Area

The image of the pressure distribution of the tire area where no damage had been produced was taken to serve as a reference for comparison with other images. Different reference profiles were also taken.

**Figure 18:**
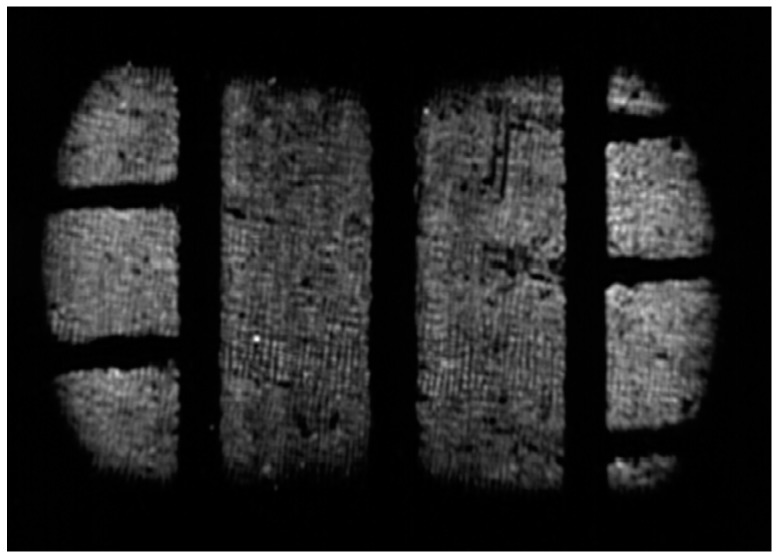
Area without defects.

**Figure 19:**
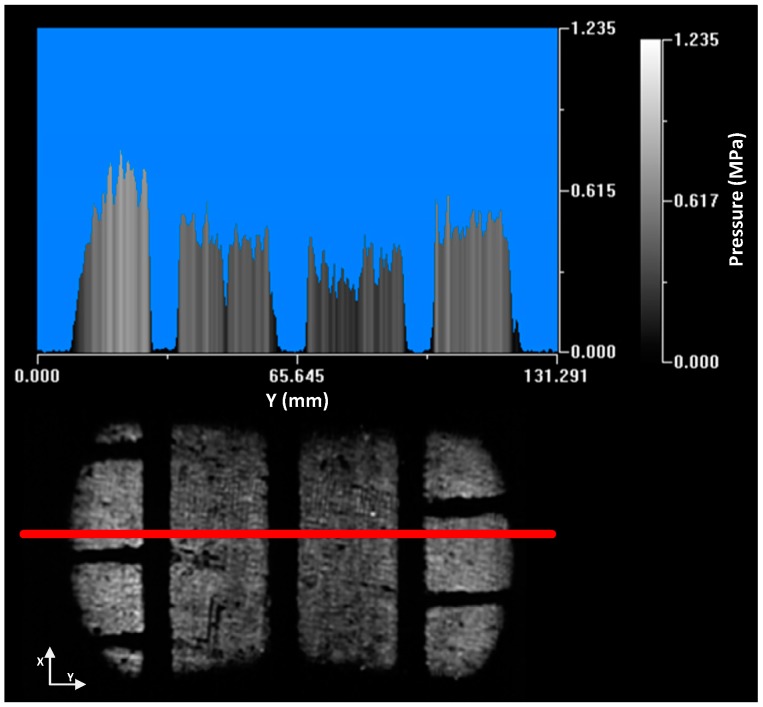
Cross profile.

It can be observed in Figure 18 that there are no areas with high grey levels. It is a homogeneous distribution in which the level of grey in the shoulders is slightly higher due to the deformation that the tire undergoes because of the load (Figure 19).

**Figure 20:**
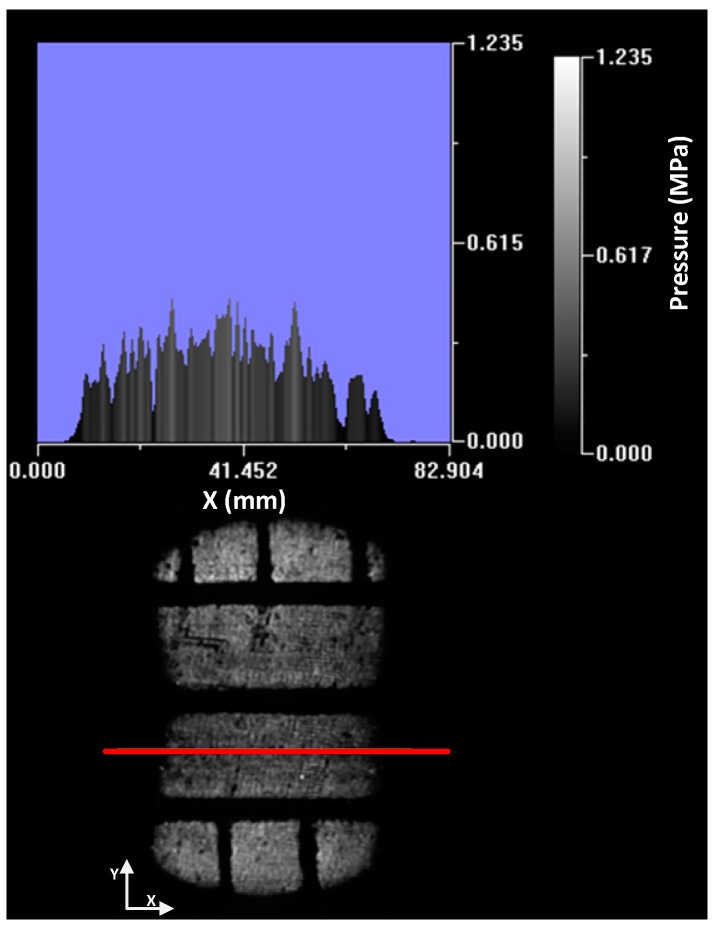
Longitudinal profile.

The pressure distribution is homogeneous in the longitudinal profile showing a smooth curve (Figure 20). The pressure in the central zone of the contact patch is higher because it is the tire area that suffers the biggest deformation of its curvature radius to adapt to the ground.

**Figure 21:**
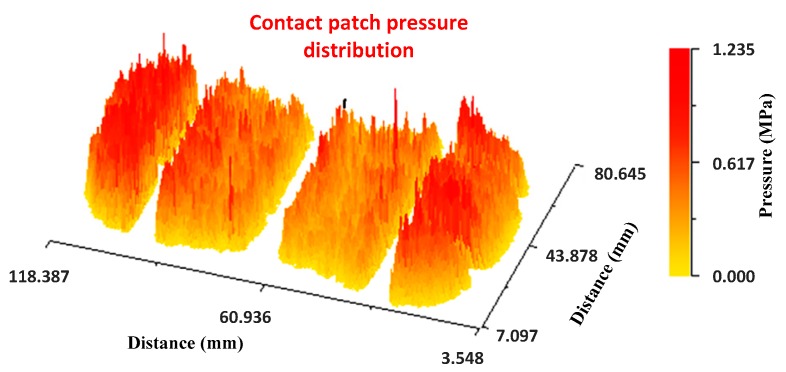
Contact patch pressure distribution. No defect area.

The pressure distribution along the entire length of the tire patch can be seen in the following image (Figure 21). As mentioned, the pressure in the shoulder zone is higher because they have to bear the load of the tire carcass.

### 5.2. Transverse CUT

**Figure 22:**
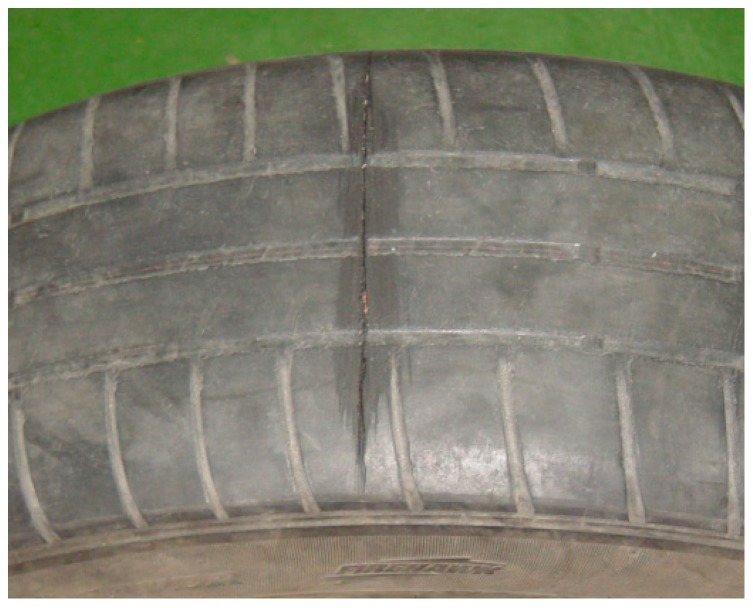
Tire damaged—transverse cut.

A transverse cut of about 4 mm deep was made to the tire, reaching the first ply of the belt, but without breaking the fibers of the casing (Figure 22).

**Figure 23:**
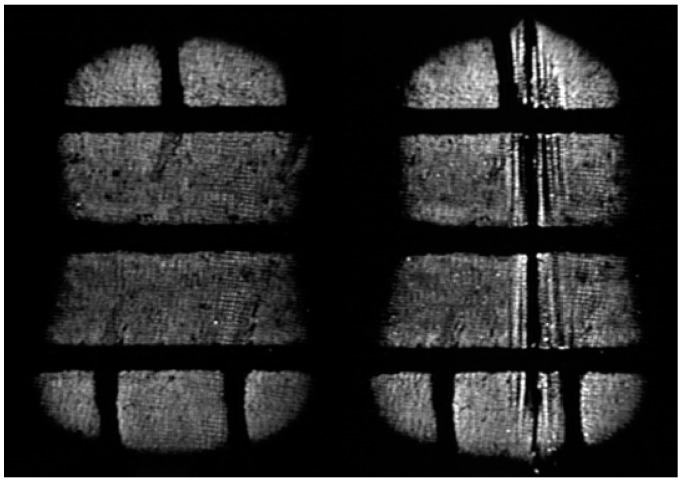
Grey levels in the contact patch: before the defect (**left**); and after the defect (**right**).

The following images show the grey levels before and after the cut was made (Figure 23).

**Figure 24:**
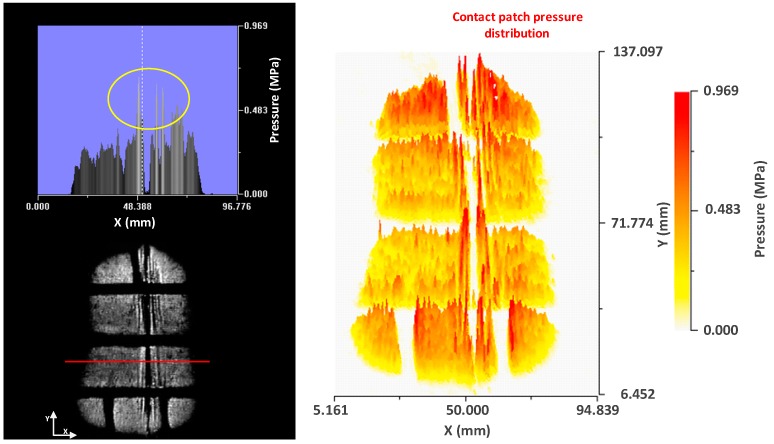
Cross profile (**left**); and contact patch pressure distribution (**right**)—transverse cut.

It can be seen how the grey levels are higher at the vertices of the cut. This means that there is a concentration of pressures at the edges of the cut. The pressure profile of a section perpendicular to the cut confirms the previous statement (Figure 24).

As mentioned, the pressure levels at the borders of the cut almost doubled with respect to the pressure levels in the rest of the patch in the profile view. A 3-D plot is included to see how this concentration of pressures is distributed along the cut.

In the 3-D representation it is observed how higher levels of grey are produced along the cut. It is also seen that some peaks appear at points where there are cuts of lower depth, although these peaks are of a lower grey level.

### 5.3. Defect Repair

**Figure 25:**
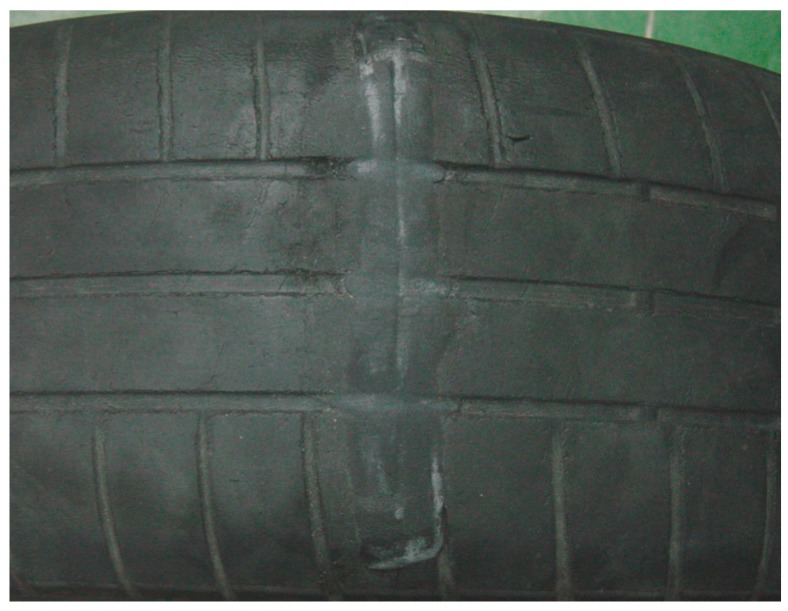
Defect repair.

The tire was repaired by using a strip of synthetic rubber and vulcanizing it. A photograph of the repaired zone is shown in Figure 25.

**Figure 26:**
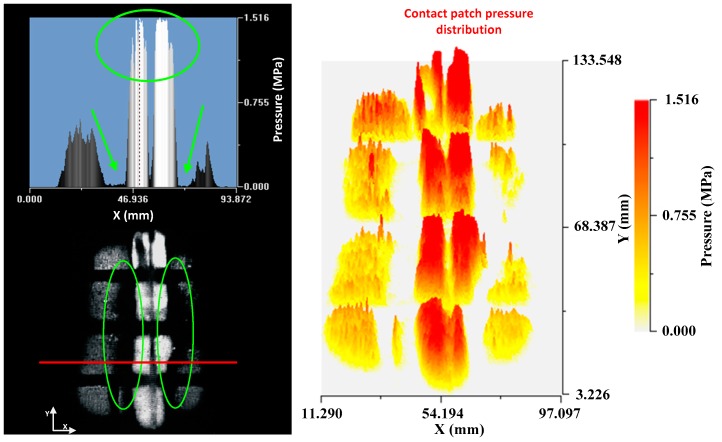
Cross-profile and pressure distribution (**left**); and contact patch pressure distribution (**right**).

The image obtained in the test bench showing the pressure distribution is included in Figure 26. The area in which the synthetic rubber was added can be clearly differentiated in the picture. There is an increase in pressure because of the added strip, which protrudes from the tread of the tire in this zone. In addition, it can be seen how the cut remains below the rubber band on the tread. Making a cross-profile shows how the pressure distribution has varied.

The cross-profile confirms the above, but it also emphasizes that a depression occurs on both sides of the supplied strip. The tire cannot be deformed enough to counteract the effect of the lifting of the strip so a hollow or cavity is generated.

The concentration of pressures produced by the rubber strip, which reaches pressure levels close to saturation, can clearly be seen in the 3-D image. Similarly, the adjacent lateral depression, where there is no tire–road contact, can be observed.

From the images obtained, it can be concluded that the technique used for the correction of the defect is not adequate since the pressure distribution is severely altered as a consequence of the addition of synthetic rubber. The modification introduced by the contribution of the material causes great loss of tire characteristics: alteration in the tread pattern which is fundamental for the correct evacuation of water and for its adherence to the ground, modification of the shape of the tire which can affects the tire balance and changes in the homogeneity of the patch. Therefore, the tire should be removed when a defect similar to the one shown in this section is present in the tire.

### 5.4. Abrasion

**Figure 27:**
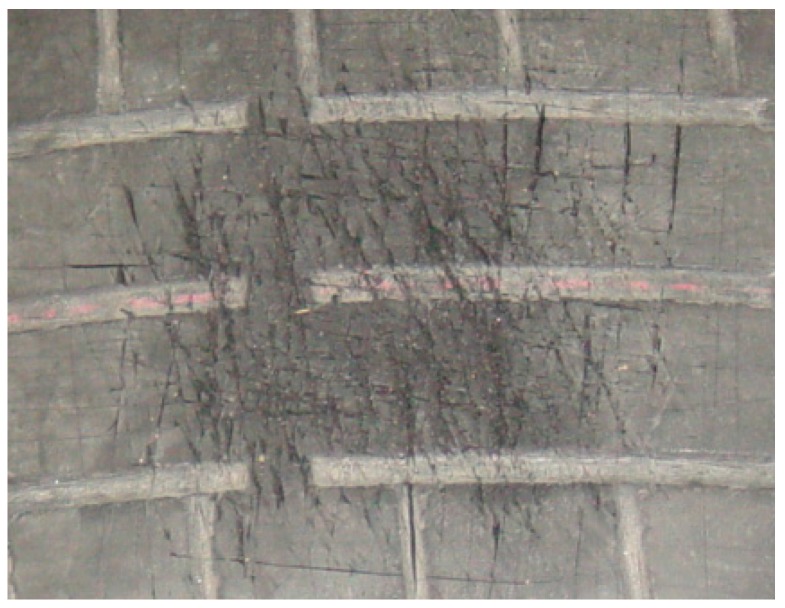
Abrasion.

The effect of abrasion was simulated in another section of the tire. This defect occurs as a result of sudden braking in which the wheels are locked. The tire quickly deteriorates as a result of slipping without rolling. This way, fragments of the tire tread are detached as a consequence of the friction and adhesion with the asphalt. Finally, a surface with a great roughness appears in the tire, as can be seen in Figure 27.

**Figure 28:**
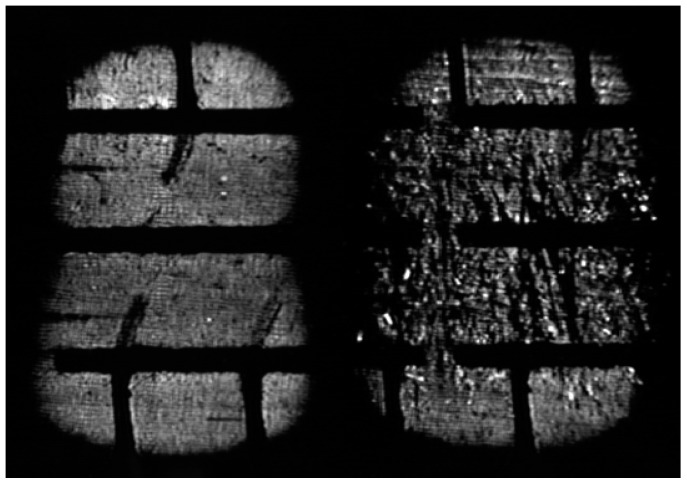
Pressure distribution: before (**left**); and after (**right**) the defect was produced.

The obtained pressure distribution is shown in Figure 28.

**Figure 29:**
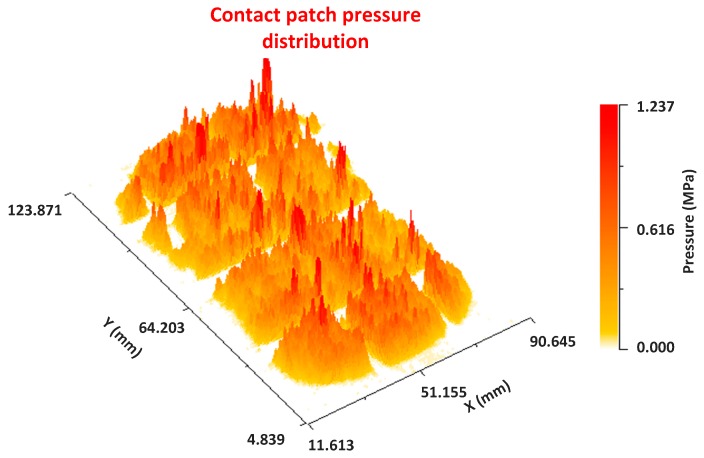
Contact patch pressure distribution—Abrasion.

An utterly random pressure map is generated. There is no established pattern and the obtained levels of grey have very different values. This effect is evident in the 3-D image where numerous spots with a high grey level appear (Figure 29).

This defect is not repaired as the material that has to be added ends up detaching because the rubber is in poor condition. A tire in these conditions does not take long to deteriorate, since the rate of material loss in the tread tends to increase which compromises the safety of the tire.

### 5.5. Long Term Parking on a Curb

The effect of improper tire contact with a non-concordant surface that compresses the tread is analyzed in this series of tests. These defects occur when the tire and the curb are in intimate contact for a long period of time. The effects of this tire misuse have already been addressed in previous studies [20], but we will expand them with new tests. Tires can become severely damaged depending on how bad the contact with the curb is.

**Figure 30:**
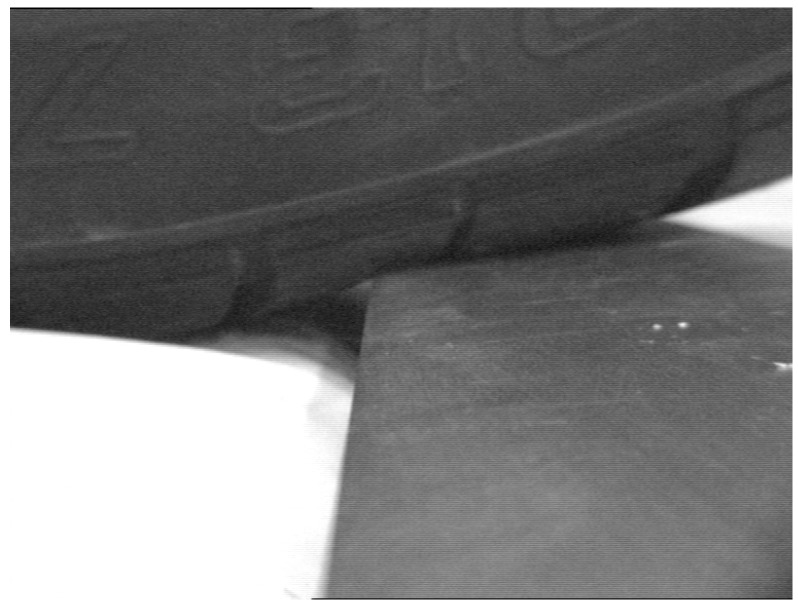
Tire support on a curb.

To simulate the support, the tire was placed on the edge of a steel part that reproduces the curb (Figure 30). Images of the pressure distribution were taken periodically to register its evolution.

**Figure 31:**
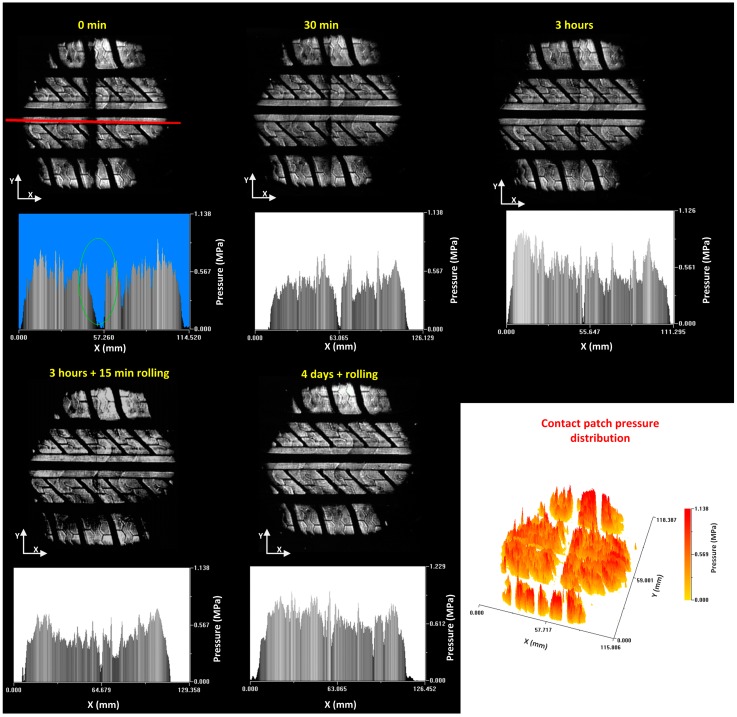
Pressure distribution, longitudinal profiles and contact patch pressure distribution.

The procedure followed is explained next. First, a 1500 N vertical load was applied to the tire for one hour with the above-mentioned support conditions. The steel part was then removed and the tire was reloaded with the same vertical load. Images were taken when the support was removed, and after 30 and 180 min in these conditions (Figure 31).

In the first obtained image, which was taken just after the piece had been removed, there is a discontinuity in the distribution of tire patch contact pressures as a consequence of the deformation of the tread. A cross profile is made to verify how this deformation influences the pressure distribution. It is observed that the deformation of the tire remains, causing an around 12 mm thickness discontinuity in the area where the tire has been in contact with the curb.

The image captured 30 min after the withdrawal of the support shows that the deformation of the tire continues although it does not have the same magnitude as the original discontinuity. The transverse profile shows how the thickness of the discontinuity is now about 4 mm wide, which shows some recovery.

The image captured after the release of the support shows a more homogeneous pressure distribution but the original defect is still perceived. The thickness of the defect has a value of around 2 mm after 3 h. Under these conditions, the tire is removed from the test machine and mounted on a machine which allows it to be rolled at speeds and load levels equal to those under normal operating conditions. The tire is rolled for approximately 15 min and the pressure distribution is measured again. The image shows that the defect still remains in the tire tread.

The profile shows a discontinuity with a thickness of between 1 and 2 mm, similar to the one that was measured before rolling it. Finally, the tire is reassembled in the machine that allows rolling the tire, but in this case the tire is kept mounted for four days and with an intermittent use. In the image obtained after this period of time there are no signs of the original deformation. The pressure distribution shows fairly homogeneous behavior, with the normal peaks due to the tread pattern. The discontinuity in the pressure map can clearly be seen in the 3-D image. This image was captured just after the defect has been produced.

## 6. Dynamic Tests

Commonly, contact patches are studied in static conditions due to the difficulties in obtaining the contact patch pressures with the tires in motion. Therefore, some effects such as front and rear tire deformation, stick and slip and tire hysteresis are not considered in this kind of studies.

This section aims to illustrate how the pressure distribution varies once the tire begins to move. Different parameters of the distribution of tire pressures are studied such as the location of the pressure center or the contact area. For this purpose, the test bench is equipped with a controlled electric motor that moves the structure that supports the glass, the rest of the system remaining static.

The tests were conducted as follows. The platform was moved with the electric motor at a very low speed (4 cm/s) causing the tire to move in pure rolling conditions. The movement was continuous. The camera and the tire remained static during the test (except for the rolling movement of the tire). The exposure time was taken into account as well as the hysteresis of the plastic lamina used in the image processing.

The most significant change that appears in the pressure distribution is the forward movement of the pressure center when the movement begins. This phenomenon occurs because the front points of the wheel are pressed by the points that have not entered the contact zone yet. The rear part of the tire is being dragged by the area of the tire which has already left the contact area. In addition, it is necessary to add the effect that causes the tractive torque that forces the pressure center to also move forward to the previous phenomenon.

**Figure 32:**
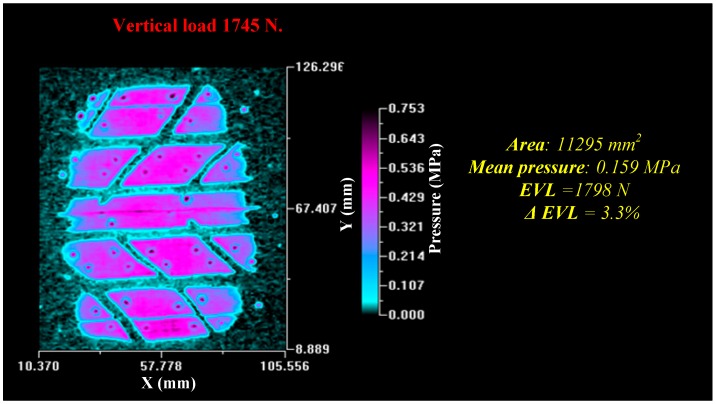
Static tire patch.

Prior to conducting the dynamic test, static samples were taken in order to compare the results to those obtained with wheel movement. The static tire patch is shown in Figure 32.

A video file was recorded to capture the entire dynamic process. From this file, some images have been selected at different time intervals (beginning, middle and final phase) to study the evolution of the pressure map.

**Figure 33:**
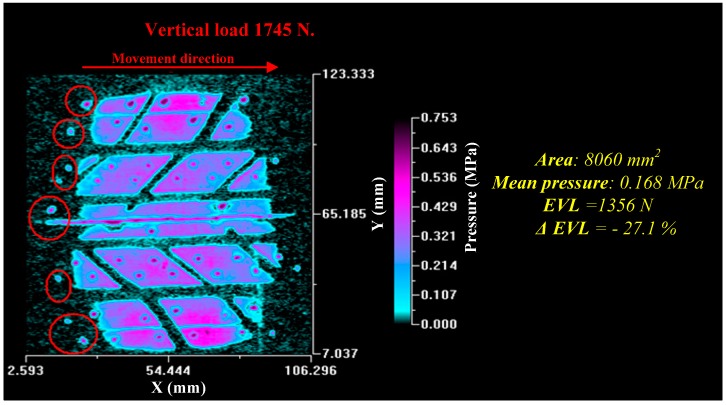
Dynamic tire patch.

It was observed that the tire generated a trail in the plastic lamina during the capture. This trail was caused by the tendency of the plastic to stick to the glass. The consequence is the marks highlighted with red circles in Figure 33.

The value of the measured area decreased. This is common behavior since the tire has a transient state in the deformation process, which does not allow the carcass to deform fully as it does in a static process. The immediate consequence is the rise in the measured mean pressure.

Doubtlessly, the most significant data are the deviation in the value of the equivalent vertical load which is around 27%. This value is very high, enough to consider the obtained numerical results incorrect. The same tests were performed with a flat tire obtaining results similar to the previous ones. These results show that there is still a great deal of research necessary to apply this technique to dynamic tests. Further experimental tests have to be carried out to calibrate the system in dynamic conditions. The work reported here is exploratory. Future works will include tests with different plastic laminas and improved image capture processes.

## 7. Conclusions

The procedure of calibration and improvement of a test bench based on the FTIR phenomenon of light has been described in this work. The test bench is used to obtain the tread characteristics and the pressure distribution in the tire–ground contact patch. A further application of the test bench is the detection of tire defects and the characterization of tire fundamental properties. The following conclusions can be drawn from the work carried out:

- Improvements have been made in the video capture system which has allowed an increase in the quality of the image obtained. Efforts have been made in the search for interface materials with a better surface texture and linear behavior in the whole range of working pressures.
- The influence of various factors on the system calibration has been evaluated. These factors are hysteresis, creep, diffuse light and crystal absorption. It has been observed that hysteresis is lower in less sensitive plastics. Besides, it has been proved that the plastics are subjected to creep, so the time of exposure to pressure must be taken into account in the calibration process. Finally, it has been found that diffuse light and the transverse absorption of light in the glass does not significantly affect the quality of the measurement.
- The following relationships between fundamental parameters are highlighted: The relationship between the actual contact area and the load is quasi linear. The contact area decreases linearly with the increase of the camber angle. The contact area decreases strongly with increasing inflation pressure and does so linearly for new tires. The pressure center moves sideways as the camber angle increases. The inflation pressure has a strong influence on the displacement of the pressure center.
- The characterization of tire defects has been studied. Defects resulting in a discontinuity of material in the tread cause a pressure increase at the edges of the discontinuity. The alterations in the pressure distribution produced by incorrect supports of the tire have a prolonged period of permanence. However, it tends to disappear with normal tire operation.
- The addition of synthetic rubber, as a technique for repairing defects in the tread, causes an imbalance in the pressure distribution by increasing the pressure in the repaired zone and diminishing the contact in its contour. This causes an uneven patch and it affects tire balance, which can lead to undesirable vibrations. Furthermore, it is appreciated how a localized loss of tire grooves takes place which reduces tire capability to evacuate water.
- Dynamic tests have been conducted. However, results show that further testing is required to calibrate the system for use in low speed tests.
